# Abnormally Low or High Ankle-Brachial Index Is Associated With the Development of Diabetic Retinopathy in Type 2 Diabetes Mellitus

**DOI:** 10.1038/s41598-017-18882-x

**Published:** 2018-01-11

**Authors:** Mei-Yueh Lee, Pi-Jung Hsiao, Jiun-Chi Huang, Wei-Hao Hsu, Szu-Chia Chen, Jer-Ming Chang, Shyi–Jang Shin

**Affiliations:** 1 Graduate Institute of Clinical Medicine, College of Medicine, Kaohsiung Medical University, Kaohsiung, Taiwan; 2 Division of Endocrinology and Metabolism, Department of Internal Medicine, Kaohsiung Medical University Hospital, Kaohsiung Medical University, Kaohsiung, Taiwan; 3 Division of Nephrology, Department of Internal Medicine, Kaohsiung Medical University Hospital, Kaohsiung Medical University, Kaohsiung, Taiwan; 4 Department of Internal Medicine, Kaohsiung Municipal Hsiao-Kang Hospital, Kaohsiung Medical University, Kaohsiung, Taiwan; 5 Faculty of Medicine, College of Medicine, Kaohsiung Medical University, Kaohsiung, Taiwan; 6 Center for Lipid and Glycomedicine Research, Kaohsiung Medical University, Kaohsiung, Taiwan

## Abstract

Although some studies have reported an association between peripheral artery disease (PAD) and diabetic retinopathy (DR) in patients with diabetes, whether or not a causal relationship exists is unknown. The aim of this study was to investigate whether PAD, as indicated by an abnormally low or high ankle-brachial index (ABI), is associated with the development of DR in patients with type 2 diabetes mellitus (DM) without DR. We enrolled 414 (221 men and 193 women) patients with type 2 DM who underwent ABI measurements at our outpatient clinic. PAD was defined as an abnormally low (<0.9) or high (≥1.3) ABI in either leg, and DR was defined as being non-proliferative or proliferative. Of the enrolled patients, 69 (16.7%) had an ABI <0.9 or ≥1.3. The median follow-up period was 23 (15–40) months, during which 74 (17.9%) patients developed DR. In multivariate analysis, an ABI <0.9 or ≥1.3 was independently associated with the development of DR (*vs*. ABI ≥0.9 to <1.3; hazard ratio, 2.186; 95% confidence interval, 1.261 to 3.789; *p* = 0.005). An abnormal ABI was associated with the development of DR in our patients with type 2 DM without DR.

## Introduction

Diabetic retinopathy (DR) is a complication caused by diabetes mellitus (DM) that affects the blood vessels of the retina and can lead to blindness. Retinopathy is characterized by a gradual progression from mild abnormalities such as increased vascular permeability to moderate and severe non-proliferative DR, which is characterized by new blood vessels growing in the retina and vitreous^1^. The annual incidence of retinopathy requiring follow-up or treatment has been reported to be 1.5%^2^, and 6% to 9% of patients with either non-proliferative or proliferative DR become blind each year^2^. The early detection of DR and early interventions such as laser photocoagulation have been shown to be effective in preventing acute visual loss. Known risk factors for the development and progression of DR include the type and duration, age, sex, body mass index, glycemic control, hypertension, nephropathy, smoking, and serum lipid levels^3–5^.

Similar to DR, peripheral artery disease (PAD), a disease of the major arteries caused by atherosclerosis, is also a vascular complication of DM^6^. In Taiwan, more than half of patients with diabetes who require an amputation reportedly have PAD^7^. The prognosis of diabetic patients who undergo lower extremity amputation is poor, with a 5-year survival rate of about 50%^8^. Although several epidemiological studies have reported an association between PAD and DR^9–12^, others have failed to identify this relationship^13,14^.

A potential association between the severity of DR and survival has been reported, primarily due to cardiovascular death in patients with diabetes^15^. This suggests that the severity of DR could be an important predictor of macroangiopathy, although whether a macroangiopathy can predict a microangiopathy in patients with diabetes is unknown. Accordingly, the aim of this study was to investigate whether an abnormally low or high ankle-brachial index (ABI) can predict the development of DR in patients with type 2 DM without pre-existing DR, and to evaluate the associated risk factors.

## Materials and Methods

### Study patients

We included all patients with type 2 DM who underwent ABI measurements in the out-patient department of a medical hospital in southern Taiwan between March 2007 and July 2015. The exclusion criteria were patients: (1) with type 1 DM (defined as those with diabetic ketoacidosis, symptoms of acute hyperglycemia with heavy ketonuria [≥3], or those who had received continuous insulin treatment in the year after the diagnosis); (2) receiving dialysis or with an estimated glomerular filtration rate (eGFR) <15 ml/min/1.73 m^2^; and (3) who had undergone a renal transplantation. Finally, 414 patients (mean age 60.9 ± 9.9 years, 221 males and 193 females) were included in this study. The study protocol was approved by the Institutional Review Board of Kaohsiung Medical University Hospital, and all participants provided written informed consent to participate in this study. The methods were carried out in accordance with the relevant guidelines.

### Assessment of ABI and brachial-ankle pulse wave velocity (baPWV)

ABI and baPWV were measured using an ABI-form device (VP1000; Colin Co. Ltd., Komaki, Japan) which measured the blood pressure in both arms and ankles^16–18^. The ABI was calculated as: ankle systolic blood pressure/arm systolic blood pressure. PAD was defined as an ABI <0.9 or ≥1.3 in either leg. For baPWV, pulse waves were obtained from both the brachial and tibial arteries simultaneously, and the transmission time was determined as the time between the initial increase in brachial and tibial waveforms. The transmission distance from the arm to each ankle was calculated according to the patient’s body height. baPWV values were computed automatically as: transmission distance/transmission time. After obtaining bilateral baPWV values, the average value was used for analysis. ABI and baPWV values were measured once per patient. The validation of this device and the reproducibility of the results have been previously reported^17^.

### Serial DR evaluations and definition of the end-point

DR was evaluated in each patient by an experienced ophthalmologist after the patient’s pupils had been dilated annually. Fluorescein angiography was performed on a case by case basis when indicated. DR was classified into three groups: non-DR, non-proliferative DR, and proliferative DR^19^. Data from these visits were retrieved for all patients from the date of ABI measurement until the development of DR. Data from at least three evaluations were available for each patient. The end-point was defined as the development of non-proliferative or proliferative DR. Follow-up period was taken as the time needed to develop DR in patients reaching the end-point.

### Collection of demographic, medical, and laboratory data

Demographic and medical data including age, sex, and comorbidities were obtained from medical records and patient interviews. The body mass index (BMI) was calculated as the ratio of the weight in kilograms divided by the square of the height in meters. Laboratory tests were conducted using fasting blood samples on an autoanalyzer (Roche Diagnostics GmbH, D-68298 Mannheim COBAS Integra 400). Levels of serum creatinine were assessed using the compensated Jaffé (kinetic alkaline picrate) method with an Integra 400 Analyzer (Roche Diagnostics, Mannheim, Germany) using a calibrator traceable in isotope-dilution mass spectrometry^20^. eGFR was calculated using the four-variable equation in the Modification of Diet in Renal Disease study^21^. Levels of urine albumin and creatinine were measured from spot urine samples using an autoanalyzer (COBAS Integra 400 plus; Roche Diagnostics, North America), and microalbuminuria was defined as a ratio of urine albumin to creatinine of ≥30 mg/g. Information regarding medications including angiotensin converting enzyme inhibitors, angiotensin II receptor blockers, β-blockers, calcium channel blockers, diuretics, statins, fibrates, insulin and oral hypoglycemia agents (OHA) during the study period was obtained from medical records.

### Statistical analysis

Statistical analysis was performed using SPSS software (version 19.0 for Windows; SPSS Inc. Chicago, USA). Data were expressed as percentage, mean ± standard deviation, or median (25^th^−75^th^ percentile) for triglyceride levels. The chi-square test was used to assess between-group differences in categorical variables, and the independent t-test was used for continuous variables. Significant variables in the univariate analysis were entered into multivariate Cox proportional hazard analysis, which was used to investigate the associations between ABI and the development of DR. Survival curves for the development of DR were obtained using the Kaplan-Meier method. A *p* value of less than 0.05 was considered to be significant.

## Results

A total of 414 patients with type 2 DM without DR were included (mean age 60.9 ± 9.9 years; 221 males and 193 females), of whom 16.7% had PAD. Comparisons of baseline characteristics between the patients with (n = 345) and without (n = 69) a normal ABI are shown in Table 1. Compared to the patients with a normal ABI (≥0.9 to <1.3), those with an abnormal ABI (<0.9 or ≥1.3) had a higher prevalence of smoking history (*p* = 0.040), higher body mass index (*p* = 0.016), higher level of glycosylated hemoglobin A_1c_ (HbA_1c_) (*p* = 0.038), lower level of high density lipoprotein (HDL)-cholesterol (*p* = 0.005), higher percentage of insulin use (*p* < 0.001) and lower percentage of OHA use (*p* = 0.024).

**Table 1:** Comparison of baseline characteristics between patients with and without a normal ABI of ≧0.9 to <1.3

Characteristics	All patients (n = 414)	ABI ≧0.9 to <1.3 (n = 345)	ABI <0.9 or ≧1.3 (n = 69)	*p*
Age (year)	60.9 ± 9.9	61.0 ± 9.3	60.8 ± 12.8	0.925
Male gender (%)	53.4	54.2	49.8	0.454
Coronary artery disease (%)	1.8	1.7	1.9	0.941
Smoking history (%)	14.5	12.9	23.0	0.040
Systolic blood pressure (mmHg)	134.4 ± 16.8	134.6 ± 16.8	133.2 ± 16.8	0.581
Diastolic blood pressure (mmHg)	79.2 ± 10.3	79.1 ± 10.3	79.5 ± 10.0	0.814
baPWV (m/s)	15.3 ± 3.1	15.3 ± 2.9	15.3 ± 3.7	0.957
Body mass index (kg/m^2^)	25.9 ± 4.1	25.6 ± 3.9	27.2 ± 4.9	0.016
DM duration (years)	9.3 ± 6.9	9.0 ± 6.7	10.6 ± 8.2	0.110
Laboratory parameters				
HbA_1c_ (%)	7.5 ± 1.7	7.5 ± 1.7	7.9 ± 1.6	0.038
Fasting glucose (mg/dL)	149.7 ± 85.3	149.3 ± 86.1	151.8 ± 81.6	0.836
Triglyceride (mg/dL)	117 (84–161)	115 (82–158)	125.5 (89.3–172.5)	0.423
Total cholesterol (mg/dL)	174.2 ± 32.7	174.2 ± 31.1	174.1 ± 40.0	0.982
HDL-cholesterol (mg/dL)	45.7 ± 11.6	46.5 ± 11.6	42.0 ± 11.2	0.005
LDL-cholesterol (mg/dL)	100.0 ± 27.8	99.9 ± 27.5	101.6 ± 29.5	0.653
eGFR (mL/min/1.73 m^2^)	87.5 ± 25.2	88.2 ± 25.4	83.8 ± 24.0	0.220
Microalbuminuria (%)	19.9	18.8	25.9	0.215
Medications				
ACEI and/or ARB use (%)	49.7	51.2	41.5	0.194
β-blocker use (%)	5.9	6.3	3.8	0.750
Calcium channel blocker use (%)	23.5	23.7	22.6	0.868
Diuretics use (%)	2.7	2.4	3.8	0.636
Statins use (%)	49.7	50.5	45.3	0.483
Fibrates use (%)	10.4	9.6	14.5	0.224
Insulin use (%)	20.3	16.9	37.7	<0.001
OHA use (%)	94.2	95.3	88.4	0.024

Abbreviations. ABI, ankle-brachial index; baPWV, brachial-ankle pulse wave velocity; HbA_1c_, glycosylated hemoglobin A_1c_; HDL, high-density lipoprotein; LDL, low-density lipoprotein; eGFR, estimated glomerular filtration rate; ACEI, angiotensin converting enzyme inhibitor; ARB, angiotensin II receptor blocker; OHA, oral hypoglycemia agent.

### The risk of developing DR

**Figure 1:**
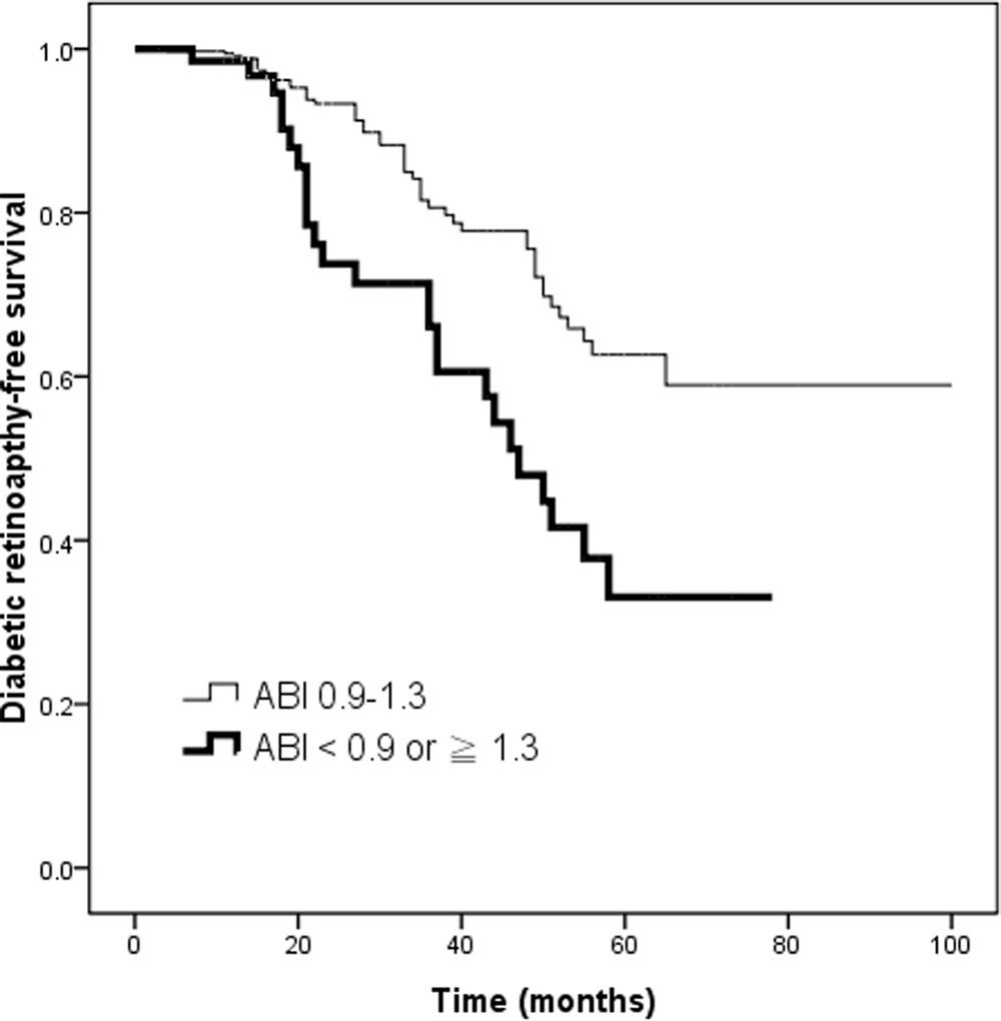
Kaplan-Meier analysis of diabetic retinopathy-free survival (log-rank *p* = 0.001) in type 2 diabetic patients subdivided according to ABI. Patients with ABI <0.9 or ≥1.3 had a worse diabetic retinopathy-free survival than those with a normal ABI ≥0.9 to <1.3.

The median follow-up period was 23 (15–40) months. During follow-up, 74 of the 414 (17.9%) patients developed DR, including non-proliferative DR (n = 70) and proliferative DR (n = 4). Table 2 shows the results of Cox proportional hazards regression analysis for the relationship between ABI and the development of DR. Univariate regression analysis showed that an ABI <0.9 or ≥1.3, smoking history, high level of HbA_1c_, albuminuria (≥30 mg/g) and insulin use were associated with increased higher risk of developing DR. In multivariate analysis, an ABI <0.9 or ≥1.3 (*vs*. ABI ≥0.9 to <1.3; hazard ratio [HR], 2.186; 95% CI, 1.261 to 3.789; *p* = 0.005), smoking history (HR, 1.981; 95% CI, 1.044 to 3.761; *p* = 0.037), high level of HbA_1c_ (per 1%; HR, 1.206; 95% CI, 1.060 to 1.373; *p* = 0.005), and albuminuria (≥30 mg/g) (HR, 1.907; 95% CI, 1.094 to 3.325; *p* = 0.023) were independently associated with the development of DR. Figure 1 illustrates the Kaplan-Meier curves for being free of DR (log-rank *p* = 0.001) according to ABI. The patients with an ABI <0.9 or ≥1.3 had worse DR-free survival than those with a normal ABI (≥0.9 to <1.3).

**Table 2:** Risk factors for diabetic retinopathy development using multivariate Cox proportional hazards model.

Parameters	Univariate		Multivariate	
	HR (95% CI)	*p*	HR (95% CI)	*p*
ABI <0.9 or ≧1.3 (*vs*. ABI ≧0.9 to <1.3)	2.280 (1.406–3.696)	0.001	2.186 (1.261–3.789)	0.005
Age (per 1 year)	1.005 (0.982–1.028)	0.698	—	—
Male (*vs*. female)	0.970 (0.612–1.535)	0.897	—	—
Hypertension	0.760 (0.440–1.313)	0.326	—	—
Coronary artery disease	4.072 (0.542–30.597)	0.172	—	—
Smoking history	2.593 (1.469–4.577)	0.011	1.981 (1.044–3.761)	0.037
Systolic BP (per 1 mmHg)	1.006 (0.991–1.021)	0.445	—	—
Diastolic BP (per 1 mmHg)	1.016 (0.989–1.044)	0.254	—	—
baPWV (per 1 m/s)	1.059 (0.971–1.155)	0.196	—	—
Body mass index (per 1 kg/m^2^)	1.041 (0.987–1.094)	0.138	—	—
DM duration (per 1 year)	0.996 (0.965–1.028)	0.816	—	—
Laboratory parameters			—	—
HbA_1c_ (per 1%)	1.277 (1.152–1.416)	<0.001	1.206 (1.060–1.373)	0.005
Fasting glucose (per 1 mg/dL)	1.002 (0.999–1.004)	0.256	—	—
Triglyceride (per log 1 mg/dL)	1.932 (0.668–5.593)	0.224	—	—
Total cholesterol (per 1 mg/dL)	0.999 (0.992–1.005)	0.687	—	—
HDL-cholesterol (per 1 mg/dL)	0.986 (0.966–1.006)	0.158	—	—
LDL-cholesterol (per 1 mg/dL)	0.999 (0.991–1.007)	0.798	—	—
eGFR (per 1 mL/min/1.73 m^2^)	1.000 (0.990–1.010)	0.972	—	—
Microalbuminuria	2.574 (1.544–4.292)	<0.001	1.907 (1.094–3.325)	0.023
Medications				
ACEI and/or ARB use	0.733 (0.425–1.265)	0.265	—	—
β-blocker use	1.437 (0.518–3.992)	0.486	—	—
Calcium channel blocker use	0.713 (0.348–1.458)	0.354	—	—
Diuretics use	1.777 (0.432–7.306)	0.425	—	—
Statins use	0.883 (0.514–1.516)	0.651	—	—
Fibrates use	0.478 (0.151–1.518)	0.211	—	—
Insulin use	2.187 (1.327–3.605)	0.002	1.400 (0.775–2.530)	0.265
OHA use	0.904 (0.364–2.244)	0.827	—	—

Values express as hazard ratios (HR) and 95% confidence interval (CI). Abbreviations are same as Table 1.

## Discussion

In this study, we evaluated the association between ABI and the development of DR in patients with type 2 DM without pre-existing DR. Our analysis showed that an abnormal ABI (<0.9 or ≥1.3) was independently associated with the development of DR. In addition, smoking, a high level of HbA_1c_ and microalbuminuria were also associated with the development of DR.

The first important finding of our study is identification of PAD, defined as ABI <0.9 or ≥1.3, as a risk factor for DR development in type 2 DM patients without DR. The ABI has been reported to be a good marker of atherosclerosis, and an ABI <0.9 has been reported to be useful in diagnosing peripheral artery occlusive disease^22–24^. In addition, an ABI ≥1.3 has been reported to indicate medial artery calcification^25^, and both of these conditions are common patients with DM^26,27^. Moreover, an abnormally low or high ABI has been reported to be a predictor of cardiovascular morbidity and mortality in patients with DM^28,29^. Previous cross-sectional studies have reported a correlation between the development of DR and an abnormal ABI^9,10,12^. Yu *et al*. reported that DR was associated with an ABI ≤0.9 in patients with type 2 DM^10^. Li also reported that DR was associated with an ABI <0.9, but not with ABI >1.3 in patients with diabetes^9^. Recently, we also showed that an abnormally low or high ABI was associated with proliferative DR in patients with type 2 DM^12^. The mechanisms underlying the association between atherosclerosis and DR are unclear. It has been hypothesized that atherosclerosis and DR share common risk factors with regards to the causal pathway^30,31^. This is supported by an apparent common mechanism underlying the development of macro- and microangiopathies in patients with type 2 DM such as obesity, insulin resistance and hypertension^32,33^. In addition, neovascularization (retinal angiogenesis) is a hallmark of proliferative DR, and angiogenesis has frequently been reported in advanced atherosclerotic lesions^34^. Besides, Blum A *et al*.^35^ have shown that compared to healthy control, type 2 DM patients with non-DR and non-proliferative DR had high levels of high-sensitivity C reactive protein and soluble vascular adhesion molecules, but decreased in patients with PDR. Biomarkers of inflammation and angiogenesis may detect the progression of diabetic vascular disease. Therefore, ABI assessments in patients with diabetes without DR may help to identify those at high-risk of developing DR.

Diabetic nephropathy is a major cause of chronic kidney disease worldwide. In Taiwan, it accounts for approximately 45% of patients with end-stage renal disease. However, the relationship between diabetic nephropathy and retinopathy is less well understood in patients with type 2 DM than in those with type 1 DM. In the present study, albuminuria (≥30 mg/g) was a risk factor for the development of DR. Retinopathy has been shown to be correlated with other complications of diabetes, probably through shared risk factors and pathways leading to vascular damage of various organs. In addition, we found that a high level of HbA_1c_ was independently associated with an ABI <0.9 or ≥1.3 and the development of DR. Along with severe hyperglycemia, high levels of reactive oxygen species and advanced glycation endproducts (AGEs) and low levels of nitric oxide lead to chronic vascular complications by altering vascular responses^36^. AGEs are known to induce migration of macrophages and T cells into the intima, promote procoagulant activity, increase vascular permeability, and impair endothelium-dependent relaxation^37^. Moreover, the inhibition of AGEs has been shown to inhibit the higher degree of atheroma associated with diabetes^38^. Glucose control may be an effective means of preventing diabetes-related micro- and macroangiopathies.

In the present study, during the median 23 months follow-up, 74 of the 414 (17.9%) patients developed DR, including non-proliferative DR (n = 70) and proliferative DR (n = 4). In Thomas RL *et al*. study^39^, the annual incidence of referable retinopathy was 0.2% in the first year, with an increase to 0.4% in the fourth year, which was lower than our study. In their study, only referable retinopathy (preproliferative or proliferative DR and maculopathy) was calculated, which might explain the inconsistent result with ours (our end-point was defined as the development of non-proliferative or proliferative DR). Another explanation may be due to the different DM duration between Thomas RL’s study (4.6 years) and out study (9.3 years). According to American Academy of Ophthalmology, DR was simply defined as people with diabetes have an eye disease. There are two main stages of diabetic eye disease or DR (1) non-proliferative DR, which also included background DR consist of microaneurysm, hemorrhage and hard exudates and (2) proliferative DR, which was mostly classified in most of the studies related to DR. Inclusion of background DR in our study, which was also considered as DR special under the classification under non-proliferative DR, makes our incidence of DR higher.

Another finding of this study is that low HDL-cholesterolemia was associated with an abnormally low or high ABI in our patients. Patients with type 2 diabetes often also have dyslipidemia, and it is a well-known risk factor for atherosclerosis and cardiovascular diseases^40,41^. High levels of low-density lipoprotein cholesterol are toxic to endothelial cells, whereas HDL-cholesterol is an important main vasoprotective lipoprotein with known vasodilatory, antioxidant, and anti-inflammatory properties, and it has also been shown to reduce the level of cholesterol in cells^42,43^. Thus, a relationship between low HDL-cholesterol and PAD is biologically plausible. Lipid-lowering agents may be effective in preventing coronary artery disease^44^, however there is no evidence that these drugs are effective in preventing or treating PAD. A meta-analysis of seven prospective randomized trials of lipid-lowering agents in patients with existing PAD reported no improvements in pain, ABI, or skin necrosis^45^. Nevertheless, despite the lack of proven treatment for PAD, most clinicians still prescribe lipid-lowering agents because of the reported benefits in reducing coronary artery and cerebrovascular diseases in patients with diabetes according to the American College of Cardiology and the American Heart Association guidelines. Further studies are warranted to develop interventions that can slow the progression of PAD in high-risk patients.

The main strength of this study is the prospective serial follow-up of data from DR evaluations over a median of 23 months in patients with type 2 DM, a group known to be at high-risk of blindness. There are also several limitations. First, mixing patients with low and high ABI values in the same group could be misleading. Those having ABI <0.9 have arteriosclerotic disease, and those having ABI ≥1.3 may have non-compressible arteries due to medial arterial calcification, which is associated to chronic renal failure and long-term diabetes. Thus, there is different pathogenesis for ABI <0.9 and ≥1.3. Furthermore, ABI is not a good marker for PAD in long-standing diabetes. The sensitivity of the ABI to correctly diagnose PAD is considerably reduced in the presence of arterial media calcification, which is associated with the presence of peripheral neuropathy^46^. Future studies using an alternative method, such as flow wave analysis, Doppler color ultrasound^47^, are needed in the presence of peripheral neuropathy. In addition, treatment with antihypertensive drugs can influence diabetic micro- and macroangiopathies, however we did not withhold any drugs during the study due to ethical considerations. Therefore, we were unable to evaluate the effect of antihypertensive drugs on diabetic micro- and macroangiopathies. To minimize the effect of this limitation, we included medication use in the multivariate analysis. Finally, treatment with statins can influence the effect of lipids including cholesterol and triglycerides. Therefore, the results associated with lipids should be interpreted with caution. However, to minimize this limitation, we included statins in the multivariate analysis.

In conclusion, our results demonstrated that an abnormally low (<0.9) or high (≥1.3) ABI was associated with the development of DR in patients with type 2 DM without pre-existing DR. Therefore, we recommend that there are possibly unifying factors in the pathogenesis of both complications and therefore more studies are needed.
